# Chronic phase CML with sole P190 (e1a2) *BCR::ABL1*: long-term outcome among ten consecutive cases

**DOI:** 10.1038/s41408-022-00696-4

**Published:** 2022-07-06

**Authors:** Maymona G. Abdelmagid, Mark R. Litzow, Kristen B. McCullough, Naseema Gangat, Animesh Pardanani, Hemant S. Murthy, James M. Foran, Rhett P. Ketterling, David Viswanatha, Kebede H. Begna, Ayalew Tefferi

**Affiliations:** 1 Division of Hematology, Mayo Clinic, Rochester, MN USA; 2 Division of Hematology, Mayo Clinic, Jacksonville, FL USA; 3 Department of Laboratory Medicine and Pathology, Mayo Clinic, Rochester, MN USA

**Keywords:** Chronic myeloid leukaemia, Chronic myeloid leukaemia

**Dear Editor**,

BCR::ABL1 oncoprotein is causally linked to both chronic myeloid leukemia (CML) and Philadelphia chromosome (Ph1)-positive acute lymphoblastic leukemia (ALL) and is the product of the *BCR::ABL1* fusion gene resulting from translocation of the 3′ region of *ABL1* oncogene from its normal position on chromosome 9q34 to the 5′ region of the *BCR* gene, located on chromosome 22q11.2. The characteristic t(9;22)(q34;q11.2) translocation occurs in ~90% of CML cases, and the derivative 22 from this translocation is recognized as the Ph1 chromosome. Most of the remaining CML cases (8–10%) represent complex 9;22 translocation with a third or fourth chromosome involved; [1] rarely, (<1%) a chromosomally “cryptic” insertional event will result in *BCR::ABL1* fusion [2]. In CML, the *BCR::ABL1* fusion transcript typically translates to the corresponding P210 (e13a2/e14a2) fusion protein with molecular weight of 210 kDa with a minority displaying other similarly oncogenic isoforms, including P190 (e1a2) and P230 (e19a2), as a result of different *BCR* breakpoints [3]. By contrast, p190 *BCR::ABL1* is the most frequent fusion transcript in Ph1-positive ALL [4]. Previously reported studies in mice have suggested similar myeloid but different lymphoid leukemogenic activity among the three aforementioned *BCR::ABL1* isoforms [3] and more recent work revealed different transcriptional and drug sensitivity profiles [5].

Two relatively large studies have provided prevalence estimates for P190 CML [6, 7]. One study included 1260 CML patients from India and reported frequencies of 60% for e14a2 (P210), 34% for e13a2 (P210), 1.2% for e1a2 (P190), 0.3% for e19a2 (P230) and other rare isoforms [6]. The second study from MD Anderson Cancer Center (MDACC) was first reported in 2009 and included 1292 CML patients and identified 14 (1%) with sole e1a2 (P190) transcripts, of which 9 were in chronic phase (CP) [7]. In an updated 2017 report [8], the MDACC group considered 2322 CML patients treated with tyrosine kinase inhibitors (TKI); 41 (1.8%) expressed e1a2 (P190) transcripts, of which 16 had blast phase (BP) and 2 had accelerated phase (AP) disease and 23 were reported to have been in CP at initial diagnosis; among evaluable CP cases, all displayed relative and absolute monocytosis while additional cytogenetic abnormalities were also more likely to be seen with P190 CML. This report suggested a prognostically detrimental effect for P190 CML in terms of higher likelihood of presenting in BP disease (39%), risk of progression from CP to BP disease (36%; more likely to be lymphoid), and inferior overall survival even after adjusting for disease phase. The objective of the current study was to review our institutional experience in CP-CML patients expressing sole *BCR::ABL1* e1a2 (P190) transcripts, in terms of overall survival, risk of blast transformation, and response patterns to TKI therapy, thus expanding upon our previous report that included only two patients with CP-CML [9].

After approval by the Mayo Clinic instituitional review board, study patients were retrospectively recruited from the Mayo Clinic institutional database, based on documentation of CML diagnosis associated with P190/e1a2 *BCR::ABL1*. Conventional criteria were used for diagnosis and disease classification as CP vs AP vs BP CML [10]. Hematologic, cytogenetic and molecular responses were assigned according to previously recommended guidelines [11]. Complete hematologic response (CHR) entailed the absence of immature myeloid cells in the peripheral blood and palpable splenomegaly, along with leukocyte count <10 × 10(9)/L, basophils <5%, and platelet count <450 × 10(9)/L. Complete cytogenetic remission (CcyR) was confirmed by karyotype or FISH and sometimes implied from a ratio of *BCR::ABL1/ABL1* transcript <1%, on the International Scale (IS)-like approach regarding measurement thresholds, as is currently applied for monitoring P210 *BCR::ABL1* transcripts. Major molecular response (MMR) entailed a ratio of *BCR::ABL1/ABL1* transcript ≤0.1%, and further considered as deep molecular response for a ratio of ≤0.01% (MR4.0), based on IS-like approach as mentioned above. Response assignments were double-checked in an independent fashion to assure accuracy. Date of last follow up or death was updated through January 2022. Overall survival indicated the interval between diagnosis and death or last follow up. Statistical analysis was based on conventional procedures using JMP Pro 16.0.0 software (SAS Institute, Cary, NC, USA).

A general CML database search for documentation of P190 (e1a2) produced 14 cases. Four of these 14 cases were excluded from the core study because of concomitant presence of P210 *BCR::ABL1* transcripts (*n* = 2; in both instances, e1a2 was a minor clone), AP disease at time of diagnosis (*n* = 1), and concomitant diagnosis of *JAK2* V617F mutation (*n* = 1). The latter patient presented with constitutional symptoms and splenomegaly and his P190 *BCR::ABL1* and *JAK2V617F* allele burdens at diagnosis were both below 1% and karyotype showed only 2 of 20 metaphases with Ph1; patient was treated with imatinib during which time his e1a2 clone was suppressed but *JAK2*V617F allele burden increased with progression to overt primary myelofibrosis, subsequently treated with allogeneic hematopoietic stem cell transplant (AHSCT); serial post-transplant mutation screening revealed disappearance of both *BCR::ABL1* e1a2 and *JAK2*V617F clones. The patient with AP-CML was treated with IFN-α initially with no response and subsequently with imatinib and achieved CCyR. At last documented follow-up, patient was alive at 12 years from diagnosis on varying doses of imatinib and going in and out of CCyR.

The current study was primarily focused on the ten patients (median age 63 years, range 50–83; 60% females) with CP-CML and harboring sole e1a2 (P190) *BCR::ABL1* (Table 1). The study patients were diagnosed between June 2006 and October 2019. At time of diagnosis; median (range) values for leukocyte count were 58 × 10(9)/L (19.3–175), hemoglobin 12.5 g/dL (10–14.4), platelet count 209 × 10 (9)/L (118–479), absolute monocyte count 7 × 10 (9)/L (0.8–29.6), monocyte percentage 12 (3–21), absolute basophil count 0.9 × 10 (9)/L (0.06–4.2), basophil percentage 2.5 (0.46–5), absolute eosinophil count 0.8 × 10 (9)/L (0.3–8.4), eosinophil percentage 2 (1–7), peripheral blood blast count 0.9 × 10(9)/L (0–4), peripheral blood blast percentage 1 (0–2), bone marrow blast percentage 1 (0–2), and bone marrow reticulin fibrosis grade 0 (0–1). All patients displayed the classic Ph1 translocation (35–100% metaphases affected; Table 1) and one patient in addition had loss of chromosome Y; no other cytogenetic abnormalities were noted at time of diagnosis, except 13q- abnormality detected by FISH only for a previous diagnosis of CLL. Quantitative P190 *BCR::ABL1/ABL1* transcript ratio at time of diagnosis was documented in 6 of the 10 patients and ranged from 26 to 50.6%; in the remaining four patients, the earliest documented pre-remission P190 transcript ratio ranged from 2.8 to 9%; none of the patients displayed P210 transcripts either at diagnosis or during follow-up.

**Table 1:** Presenting features and clinical course information among ten patients with chronic phase chronic myeloid leukemia (CP-CML) harboring sole *BCR::ABL1* e1a2 (P190) transcripts.

Variables	All patients (*n* = 10)
Age in years at diagnosis; median (range)	63 (50–83)
Females, *n* (%)	6 (60)
Leukocytes × 10^9^/L; median (range)	58 (19.3–175)
Absolute monocyte count × 10^9^/L; median (range)	7 (0.8–29.6)
Blood monocyte %; median (range)	12 (3–21)
Absolute eosinophil count × 10^9^/L; median (range)	0.8 (0.3–8.4)
Blood eosinophil %; median (range)	2 (1–7)
Absolute basophil count × 10^9^/L; median (range)	0.9 (0.06-4.2)
Blood basophil %; median (range)	2.5 (0.46-5)
Absolute myelocyte count × 10^9^/L; median (range)	3.3 (0-29.7)
Blood myelocyte %; median (range)	5.5 (0-3)
Blood blast count × 10^9^/L; median (range)	0.9 (0-4)
Blood blast %; median (range)	1 (0-2)
Bone marrow blast %; median (range)	1 (0–2)
Bone marrow reticulin fibrosis grade; median (range)	0 (0-1)
Hemoglobin g/dL; median (range)	12.5 (10–14.4)
Platelets × 10^9^/L; median (range)	209 (118–479)
Philadelphia chromosome karyotype at time of diagnosis, *n* (%)	100%
No. of metaphases analyzed; median (range)	20 (20–25)
Metaphases involved, (*n*)	
100%	7
80%	1
35%	2
Palpable splenomegaly, n (%)	2 (20)
Symptoms at time of diagnosis seen in 2 or more patients:	
Asymptomatic, *n* (%)	5 (50)
Fatigue, *n* (%)	3 (30)
Abdominal discomfort, *n* (%)	2 (20)
Tyrosine Kinase Inhibitor response at any time:	
Complete hematologic response (CHR), *n* (%)	10 (100)
Complete cytogenetic response (CCyR), *n* (%)	8 (80)
Major molecular response (MMR), *n* (%)	5 (50)
Follow-up period from diagnosis in years; median (range)	6 (1–16)
Deaths, *n* (%) *Both deaths were unrelated to CML*	2 (20)
Progression to Acute Myeloid Leukemia, *n* (%)	0 (0)
Allogeneic hematopoietic stem cell transplant, *n* (%)	0 (0)

At a median follow-up of 6 years from diagnosis (range 1–16), two (18%) deaths were reported, both from causes unrelated to CML. In addition, there were no instances of disease transformation into AP-CML or BP-CML or AHSCT. Figure 1 illustrates treatment details, response patterns, and follow-up durations. All 10 (100%) patients had achieved CHR, 8 (80%) CCyR, and 5 (50%) MMR, following treatment with TKI. Median (range) time to achieve CHR was 4 months (3–24), CCyR 12 months (8–27), and MMR 37 months (17–92). In all, 8 (80%) patients started treatment with imatinib and all 8 achieved or maintained CHR while imatinib-induced CCyR and MMR were documented in 2 (25%) and 1 (13%) patients, respectively; the only patient achieving MMR with imatinib was on 600 mg/day dose (Fig. 1). Six patients received nilotinib with CCyR and MMR rates of 50 and 33% while 5 patients received dasatinib with CCyR rate of 40%; one patient received bosutinib and achieved MMR (Fig. 1). As illustrated in Fig. 1, all 4 patients followed for at least 8 years had achieved MMR; 1 on imatinib, 2 on nilotinib and one on bosutinib. Patient 5 on Fig. 1 died from CML-unrelated cause at age 84 years with multiple comorbidities including heart failure, coronary artery disease and severe peripheral vascular disease. Patient 6 on Fig. 1 also died of CML-unrelated cause at age 89 years, with multiple comorbidities including severe dementia, heart failure, and aortic stenosis, after being on treatment for 4 years and had achieved nilotinib-induced CCyR at time of death. Next-generation sequencing and *ABL* kinase domain mutation studies were not consistently performed but a G250E *ABL* kinase domain mutation was documented in patient 6 (Fig. 1) who was switched from imatinib (CHR) to nilotinib (CCyR).

**Fig. 1:**
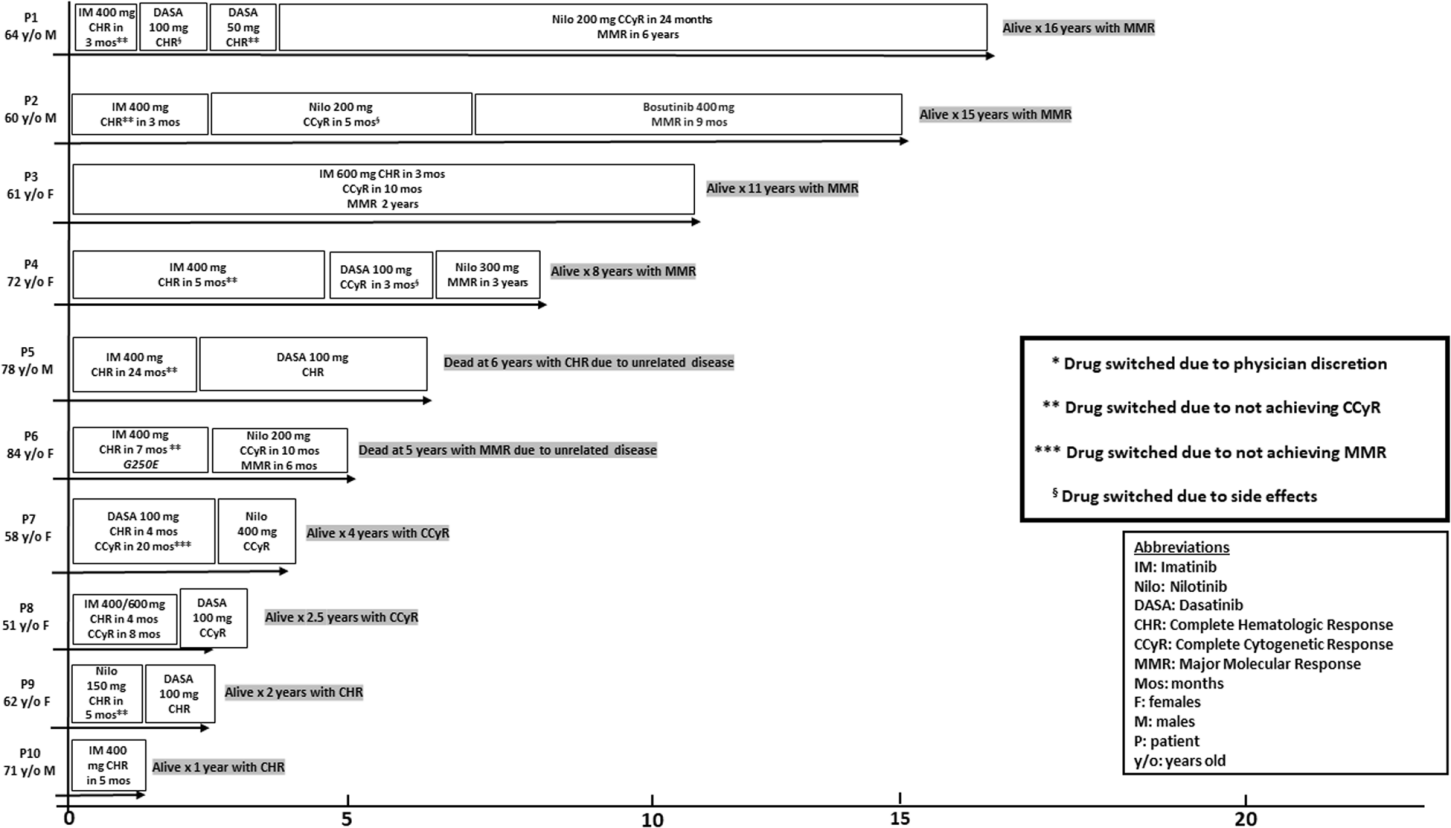
Treatment history, maximal response, time to response, and follow-up time among ten Mayo Clinic patients with chronic-phase chronic myeloid leukemia associated with *BCR::ABL1* e1a2 (P190) transcript.

Observations from the current study paint a relatively better picture, than previously suggested [8], on the outlook of CP-CML patients with sole e1a2 (P190) *BCR::ABL1* transcripts. None of our 10 CP-CML patients progressed into BP-CML, after a median follow-up of 6 years; by contrast, 9 (36%) of 25 MDACC patients with either CP-CML (*n* = 23) or AP-CML (*n* = 2) were previously reported to have experienced blast transformation, after a median follow-up of 7.5 years [8]. Additional cytogenetic abnormalities at diagnosis were reported in the MDACC [8] but not in the current study, thus partly explaining the discrepancy in blast transformation rate between the two cohorts. The Mayo Clinic and MDACC experience was otherwise consistent regarding distinct presenting features, including older age distribution, monocytosis and the slower pace of achieving CCyR or MMR, as well as the apparently lesser efficacy of imatinib in inducing MMR. The number of informative cases were too small and treatment schedule too heterogeneous to comment on comparative efficacy of the different TKIs employed. However, the apparent additional value from 2nd generation TKIs, noted in the current study, promises the prospect of deeper and faster responses from 3rd generation TKIs, including ponatinib and asciminib.

## Data Availability

Please email the corresponding author.
